# A comparative study on the pharmacological effects of Achyranthes root against atherosclerosis in ApoE^−/−^ mice based on the spectrum-effect relationship

**DOI:** 10.1186/s42826-026-00288-4

**Published:** 2026-07-10

**Authors:** Xiaoming Zou, Jie Su, Bingbing Gao, Shanshan Lei, Xiongying Li, Bo Li

**Affiliations:** 1 The First Affiliated Hospital of Zhejiang Chinese Medical University (Zhejiang Provincial Hospital of Chinese Medicine), Hangzhou, Zhejiang 310006 P.R. China; 2 Zhejiang Chinese Medical University, Hangzhou, Zhejiang 310053 P.R. China; 3 The Second Affiliated Hospital of Zhejiang Chinese Medical University, Hangzhou, Zhejiang 310007 P.R. China; 4 Tongde Hospital of Zhejiang Province Affiliated to Zhejiang Chinese Medical University (Tongde Hospital of Zhejiang Provinceï¿¿, Hangzhou, Zhejiang 310000 P.R. China; 5 Fuzhou Medical University, Fuzhou, Jiangxi 344100 P.R. China

**Keywords:** Achyranthes root (Niu Xi), Atherosclerosis, Spectrum-effect relationship, SDF-1/CXCR4

## Abstract

**Background:**

In traditional Chinese medicine, herbal processing is used to modify the effects of herbs. *Achyranthes bidentata* BL (Niu Xi) is commonly used to treat atherosclerosis and cardiovascular diseases, in both raw (RC) and salted (SRC) forms. Salting is known to enhance its effects, particularly on kidney function, but the differences in anti-atherosclerosis efficacy between RC and SRC have not been studied.

**Results:**

A total of 491 and 501 chemical components were identified in RC and SRC, respectively, with 337 shared components. RC at a high dose (2.4 g/kg/d) was more effective against AS than the same dose of SRC over 12 weeks. Both RC and SRC increased SDF-1, SCF-1, and CXCR4 levels in the aorta of model mice compared to controls, suggesting these agents may protect the vascular endothelium through a related signaling axis. A gray relational analysis of 19 common components using UPLC-MS peak areas showed correlations (gray relational value > 0.6) with serum total cholesterol and bone marrow SDF-1 levels.

**Conclusions:**

Both RC and SRC have anti-AS effects, but salt-processing does not improve this activity.

**Supplementary Information:**

The online version contains supplementary material available at 10.1186/s42826-026-00288-4.

## Background

Atherosclerosis (AS) is a chronic disease with complex pathology. It can lead to the onset and development of cardiovascular and cerebrovascular diseases, including peripheral artery disease and multi vessel disease, potentially resulting in death [1]. As the global population continues to skew older and as unhealthy lifestyles become increasingly common, the incidence and mortality of cardiovascular and cerebrovascular diseases are increasing. According to the American Heart Association’s Heart Disease and Stroke Statistics of 2022, the global mortality associated with cardiovascular diseases increased by 18.7% from 2010 to 2020. Over that time period, the global burden of risk factors, disease, and injury related to cerebrovascular issues increased by 70.0%, and the number of stroke deaths increased by 43.0% between 1990 and 2019. Strikingly, the incidence and death related to stroke have even begun to trend higher among younger populations [2, 3]. The prevention and treatment of cardiovascular and cerebrovascular diseases are thus becoming increasingly important. At present, the therapeutic options are limited, and it is of great clinical significance to find new therapeutic targets and effective drugs.

The main risk factors of AS include dyslipidemia, endothelial cell injury, inflammation, and immune dysfunction, among which vascular endothelial cell injury is considered to be the main trigger for the occurrence and development of the disorder [4]. Endothelial progenitor cells (EPCs) are precursor cells that can cycle, proliferate, and differentiate into mature vascular endothelial cells. They play a key role in the process of angiogenesis and endothelial repair, and they maintain the stability of the intravascular environment [5, 6]. Stimulated by physiological or pathological factors, EPCs can be mobilized from bone marrow to peripheral blood to participate in the repair of damaged blood vessels [5, 6]. When the migration function of EPCs is impaired, the body’s ability to repair the vascular endothelium declines, greatly increasing the risk of AS [7]. Conversely, enhancing the ability of EPCs to repair the vascular endothelium represents an attractive potential mechanism for clinical interventions in the occurrence and development of AS [8, 9].

The dry root of *Achyranthes bidentata* BL, a flowering plant of the family Amaranthaceae, has been used extensively in traditional Chinese medicine due to its effects of removing blood stasis, dredging meridians, and tonifying the liver and kidney. Historically, *Shennong Materia Medica Classic*, written within the first two centuries of the Common Era, recognized the utility of this material in “chasing blood and Qi.” Modern pharmacological studies have shown that extracts of *A. bidentata* have anti-inflammatory, anti-oxidative stress, anti-osteoporosis, anti-aging, and lipid-regulating effects [10–12]. Network pharmacology approaches have emphasized the anti-inflammatory and lipid-lowering effects of this material, and they have indicated that *A. bidentata* extracts may improve vascular endothelial function via multiple mechanisms and by interacting with multiple targets, thus potentially serving as a valuable preventative or therapeutic agent for AS [13].

The activities of materials derived from *A. bidentata* have been found to depend on the processing of the plant. Raw *A. bidentata* (RC) is a commonly used form, but salted *A. bidentata* (SRC) is also widely used. SRCis produced through a process that involves cutting RC into segments, briefly soaking these segments in salt water, heating to apparent dryness, and then sun-drying to remove remaining moisture. While SRC has been applied to multiple clinical applications, any differences in the therapeutic effectsof RC and SRC in the context of AS have not yet been established.

The ability of *A. bidentata* to exert an anti-AS role by protecting against vascular endothelial injury has been attributed to several effective components [14], and changes to these components during processing of RC into SRC may underlie the differences in these preparations. To address such issues, spectrum-effect relationship analysis can be used to statistically connect chemical profiles with pharmacological outcomes. This process can link bioactive components to therapeutic effects; identification of such links is particularly useful for traditional Chinese medicines that consist of multicomponent systems that are often exceedingly complex [15].

In the present study, the bioactive components of RC and SRC were identified usingan UPLC-MS-based strategy. The efficacies of RC and SRCpreparations in the treatment of a mouse model of AS were evaluated by investigating the treatments’ effects on blood lipid levels, aortic root tissue lesions, and lipid deposition. Quantification of endothelial progenitor cells in the peripheral blood was also used as a means to evaluate treatment efficacy in the mouse model. On a molecular level, we analyzed the expression of markers of AS, specifically stromal cell-derived factor-1 (SDF-1) and C-X-C chemokine receptor type 4 (CXCR4), in the mouse aorta. The overall identification of bioactive components in RC and SRC was performed using grayscale correlation analyses to compare the chemical compositions of the formulations to the observed pharmacological effects.

## Methods

### Preparation of RC and SRC

RC and SRC were purchased from Zhejiang University of Traditional Chinese Medicine Traditional Chinese Medicine Decoction Co., Ltd. RC (lot 221102) was purchased from Zhejiang Chinese Medical University Traditional Chinese Medicine Pieces Co., Ltd. SRC (lot 20221129) was purchased from Hangzhou Hua dong Traditional Chinese Medicine Pieces Co., Ltd. RC and SRC were prepared by decocting the powder in boiled water at a ratio of 1:10 for 2 h, and the resulting material was concentrated to equivalent to 4 g of crude drug per mL for storage. Appropriate dilutions were performed immediately prior to administration into animal models.

### UPLC-MS analysis

Samples (0.1 g) of RC or SRC freeze-dried powder were suspended in 1000 µL of 80% methanol. Glass beads were added and then the samples were ground for 5 min and mixed by vortexing for 10 min. The clarified supernatant was recovered by centrifugation for 10 min at 4 ℃ and 20,000*g*. The chemical components were analyzed by UPLC-MS using a Q-Orbitrap system precisely as described previously [14]. An analysis of the chemical components of RC and SRC was performed using a Thermo Scientific™ Q Exactive high-resolution mass spectrometer coupled with an UltiMate 3000 RS UHPLC system. Chromatographic separation was carried out on a Welch AQ-C18 column (2.1 × 150 mm, 1.8 μm) maintained at 35 °C. The mobile phase consisted of (A) 0.1% formic acid in water and (B) methanol. The gradient elution program was set as follows: 0–1 min, 2% B; 1–5 min, 20% B; 5–10 min, 50% B; 10–15 min, 80% B; 15–20 min, 95% B; 20–27 min, 95% B; 27–28 min, 2% B; 28–30 min, 2% B. The flow rate was 0.3 mL/min, the injection volume was 50 µL, and the auto sampler temperature was set at 10 °C. Mass spectrometry was performed in both positive and negative electrospray ionization (ESI) modes. The key ion source parameters were: spray voltage, 3.2 kV (for both positive and negative modes); capillary temperature, 300 °C; sheath gas pressure, 40 arbitrary units; auxiliary gas pressure, 15 arbitrary units; and probe heater temperature, 350 °C. The full scan mass range was set to *m/z* 100–1500 with a resolution of 70,000 (full mass). Data acquisition was performed using Full MS/dd‑MS² mode, and the MS/MS resolution was set to 17,500. The raw data acquired by high‑resolution LC‑MS were preliminarily processed using CD 3.3 (Compound Discoverer 3.3, Thermo Fisher), followed by database matching against the mzCloud library.

### Animals and study design

Thirty-six male ApoE^−/−^C57BL/6J mice (20 ± 2 g) and six male wild-type C57BL/6J mice (18 ± 2 g) were obtained from GemPharmatech Co.,Ltd (license number: SCXK (su) 2018-0008, Jiangsu, China). The mice were maintained in a facility with a temperature range of 22 to 26 °C anda humidity range of 50 to 60%, and the mice consistently experienced 12 h light/dark cycles. Food and water were provided ad libitum. Mice were adapted for 7 d prior to experimentation.

The six wild-type mice were set as the normal control group (NG) and were maintained on normal mouse chow. The 36 ApoE^−/−^ mice were fed a high-fat diet (Boaigang Biological Technology Co., Ltd., BIOG109C) for 12 weeks and then divided into six groups according to body weight. The ApoE^−/−^ mice in the six experimental groups were treated as follows: animals in groups RC-H and SRC-H were administered high doses (2.4 g/kg/d) of RC or SRC, respectively; animals in groups RC-L and SRC-L were administered low doses(1.2 g/kg/d) of RC or SRC, respectively; animals in positive group (atorvastatin calcium group, LG) were administered 2 mg/kg/d; and animals in the model group (MG) were administered normal saline, as mice in NG. RC, SRC, and normal saline were oral administered once daily for 12 weeks. Summary of the experimental protocol in ApoE^−/−^mice were shown in Figure S1.

### Bodyweight measurements and blood analyses

After giving the 12 weeks of treatment, the mice were weighed and then fasted overnight. Blood drawn from the ophthalmic venous plexus was centrifuged at 3500 rpm for 10 min to collect serum. The serum levels of total cholesterol(TC), high-density lipoprotein cholesterol (HDL-c), low-density lipoprotein cholesterol (LDL-c), and triglycerides (TG) were measured with corresponding biochemical kits using an automatic biochemical analyzer (HITACHI-7020, Japan). Aliquots of approximately 100 µL of blood collected from the retinal venous plexus into tubes containing EDTA anticoagulant were subjected to complete blood count testing using a BC-500 blood cell analyzer (Mairui).

### Echocardiography

Echocardiographic assessments were conducted prior to euthanasia to evaluate cardiac function. The mice were positioned supine on a plastic board, and the fur on the anterior thoracic region was removed. Anesthesia was induced using isoflurane inhalation at a concentration of 2%, combined with 70% oxygen. Images were acquired utilizing a ZC3 SCI Small Animal Ultrasound Imaging System(Shenzhen Mindray Bio-Medical Electronics Co., Ltd). M-mode echocardiographic recordings were initially obtained at the level of the mitral valve chordae. Next, echocardiography was used to measure the left ventricular short-axis shortening rate, heart rate (HR), interventricular septum fractional thickening percentage (IVS), stroke volume (SV), left ventricular end-diastolic interventricular septal thickness (IVSd), left ventricular posterior wall thickness (LVPW), and ejection fraction (EF). These parameters were continuously monitored across three cardiac cycles, and the results were averaged.

### Oil red O staining

After 12 week experimental period and after blood collection, the mice were sacrificed by cervical dislocation. The entire aorta, including the aortic arch, the thoracic aorta, and the heart, was harvested. The tissue was cut open longitudinally about the aortic arch and thoracicaorta to expose the atherosclerotic plaques, then fixed overnight in 4% formaldehyde at 4 °C. Aortic root sections were prepared using afreezing microtome. These tissues and thin sections were washed three times in PBS with a 10-min interval between washes and then rinsed in 60% isopropanol. The samples were stained with oil red O (Haoke, HK2032R) for 15 min, rinsed with 60% isopropanol until the background became transparent, and then mounted with gelatin on cover slides for long-term storage. Images were recorded using a light microscope.

### Histopathology

Histopathological staining was performed according to previous studies with slight modifications [16]. Aorta tissues were fixed with 10% formalin and then embedded in paraffin. Samples were cut into 5 mm sections and fixed on a glass slide, followed by staining with hematoxylin-eosin (H&E). Due to sample loss during processing, the final number of quantifiable samples in each processing group was three. The percentage of plaque area was determined using Image J and calculated by expressing the plaque area relative to the total vascular area.

### ELISA

To prepare samples of bone marrow supernatant, the femurs and tibias were harvested from mice following sacrifice, and the surrounding skin and muscle tissues were removed. The lumens of the femurs and tibias were rinsed with PBS, and the rinse solution was centrifuged at 100 rpm for 5 min. The supernatant was stored at -80 °C prior to subsequent analyses. Corresponding ELISA kits (BEINGLAY) were used to measure the levels of SDF-1 (cat # MU30235) and Stem Cell Factor (SCF-1, cat # MU30369) in serum and bone marrow supernatant samples, according to the manufacturer’s instructions.

### Flow cytometry and cell sorting

Place the collected bone marrow cell suspension in an EP tube and centrifuge at 1000 revolutions per minute for 5–10 min. Discard the supernatant, then lyse the red blood cells with a red blood cell lysis buffer until the solution becomes a clear red color, which takes approximately 20 min. Add cell washing buffer (BD Pharmingen, cat#554656), centrifuge at 1000 revolutions per minute for 6 min, discard the supernatant, and wash the cells 1–2 times in this manner, discarding the supernatant each time. For the sample testing tube, add 1 µl of fluorescently labeled antibody FITC Rat Anti-Mouse Ly-6 A/E(BD Pharmingen, cat#557405); PE Rat Anti-Mouse FLK-1(BD Pharmingen, cat#560070); PE Rat Anti-Mouse CD117(BD Pharmingen, cat#553355), gently blow to mix uniformly. Incubate in the dark at 4 °C for 30 min, then add cell washing buffer, centrifuge at 1000 revolutions per minute for 6 min, discard the supernatant, and wash the cells 2–3 times in this manner. Resuspend the cells in 300 µl of cell washing buffer, and detect CD117^+^/Ly-6^+^ and FLK^+^/Ly-6^+^ cells using flow cytometry and count them.

### Immunohistochemical staining

Immunohistochemical analyses were performed essentially as described previously [17]. Briefly, paraffinsections wereincubated with primary antibodies recognizing mouse CXCR4 (cat # YN5620, Immunoway) and SDF-1 (cat # YT4225, Immunoway) and then with a horseradish peroxidase-conjugated goat anti-rabbit IgG secondary antibody (cat # PV-6001, Zhongshan Goldenbridge Biotechnology Co. Ltd, Beijing, China). The signals were visualized by stainingwith 3,3’-diaminobenzidine, and the nuclei were counterstained with hematoxylin. Positive stainingpresented as a yellow color under an ECLIPSE Timicroscope (Nikon, Japan), and quantitative analyses of staining was performed using ImageJ.

### Comprehensive weight analysis

Comparisons of the efficacy of RC and SRC in the ApoE^-/-^ mouse model were performed essentially as previously described [18]. In short, I level index (including TC, TG, HDL, LDL and plaque area) and II level index (Gran%(%), Lym%(%), Mon%, WBC, Mon, HR, FS, IVS, SV, IVSD, LVPW%) were first established. Then, based on the results of each indicator, weighted 0.7:0.3, evaluation scores and grades were calculated.

For evaluation rating, when comparing the treatment group with the model group, the therapeutic efficiency (V) value=(Y-M)/M×100, where Y is the value of each treatment group indicator, and M is the corresponding value of the model group. For negative indicators, V value was calculated as (M-Y)/M×100.

Index scores were calculated for each treatment group as comprehensive weighted evaluation scores (P) using Eq. (1), in which m is the number of significant effects; n is the number of effective cases; p is the number of relatively effective cases; and q is the number of ineffective cases.

1 $$\eqalign{ P{\text{ }} = {\text{ }} & \left[ {0.7 \times \left( {0.4 \times m\, + \,0.3 \times n\, + \,0.2 \times p\, + \,0.1 \times q} \right)} \right]{\text{ }} \cr + {\text{ }} & [0.3 \times \left( {0.4 \times m\, + \,0.3 \times n\, + \,0.2 \times p\, + \,0.1 \times q} \right] \cr} $$

Note: “m” is the number of significant index (V value > 25); “n” is the number of valid factors (V value > 20); “p” is the number of relatively valid factors (V value > 15); and “q” is the number of invalid factors (V value > 13).

The comprehensive score P was used to evaluate the assessment objects. If P was greater than or equal to X%, the comprehensive weight grade was considered “good”; if P was between X and Y%, the comprehensive weight grade was considered “medium”; if P was less than Y%, the comprehensive weight grade was considered “average.” The values of X% and Y% were calculated as shown in Eq. (2) and Eq. (3), respectively, where n_p_ is the number of primary indicators, and n_s_ is the total number of secondary indicators.

2 $$X{\text{ }} = {\text{ }}\left[ {\left( {{n_p} \times {\text{ }}0.4{\text{ }} \times {\text{ }}0.7} \right){\text{ }} + {\text{ }}\left( {{n_s} \times {\text{ }}0.4{\text{ }} \times {\text{ }}0.3} \right)} \right]{\text{ }} \times {\text{ }}0.85{\text{ }} \times {\text{ }}100$$

3 $$Y{\text{ }} = {\text{ }}\left[ {\left( {{n_p} \times {\text{ }}0.4{\text{ }} \times {\text{ }}0.7} \right){\text{ }} + {\text{ }}\left( {{n_s} \times {\text{ }}0.4{\text{ }} \times {\text{ }}0.3} \right)} \right]{\text{ }} \times {\text{ }}0.6{\text{ }} \times {\text{ }}100$$

Note: n_p_: Total number of I-level indicators. n_s_: Total number of II-level indicators.

### Grayscale correlation analysis

Correlations between drug components and efficacy indicators were determined by first finding the areas of peaks representing drug components in the UPLC-MS analysis. These areas are referred to as common peak areas in investigations of drug components. Dimensionless processing of the peak areas and efficacy indicators was performed using normalization. The dimensionless value Y_ij_ was calculated using Eq. (4), where the minimum and maximum values in the raw data X_jj_ are represented as X_min_ and X_max_, respectively.

4 $$\:{Y}_{jj}=\:\frac{{X}_{jj}-\:{X}_{min}}{{X}_{max}-\:{X}_{min}}$$

Subsequently, the SPSSPRO platform (https://www.spsspro.com/) was used to analyze correlations between efficacy indicators and common peaks, essentially as previously described [15]. Lipid levels and plaque areaswere taken as the parent sequences, while the common peak area was taken as the subsequence. The degrees of correlation were calculated, with a higher correlation degree indicates a stronger association between the component and efficacy indicator.

### Statistical analysis

Data are reported as mean ± SDand analyzed using SPSS 16.0. Student’s t-tests or ANOVA with LSD-t test were used for group comparisons, with *p* < 0.05 considered to indicate significance.

## Results

### The components of RC and SRC as determined by UPLC-MS

**Fig. 1:**
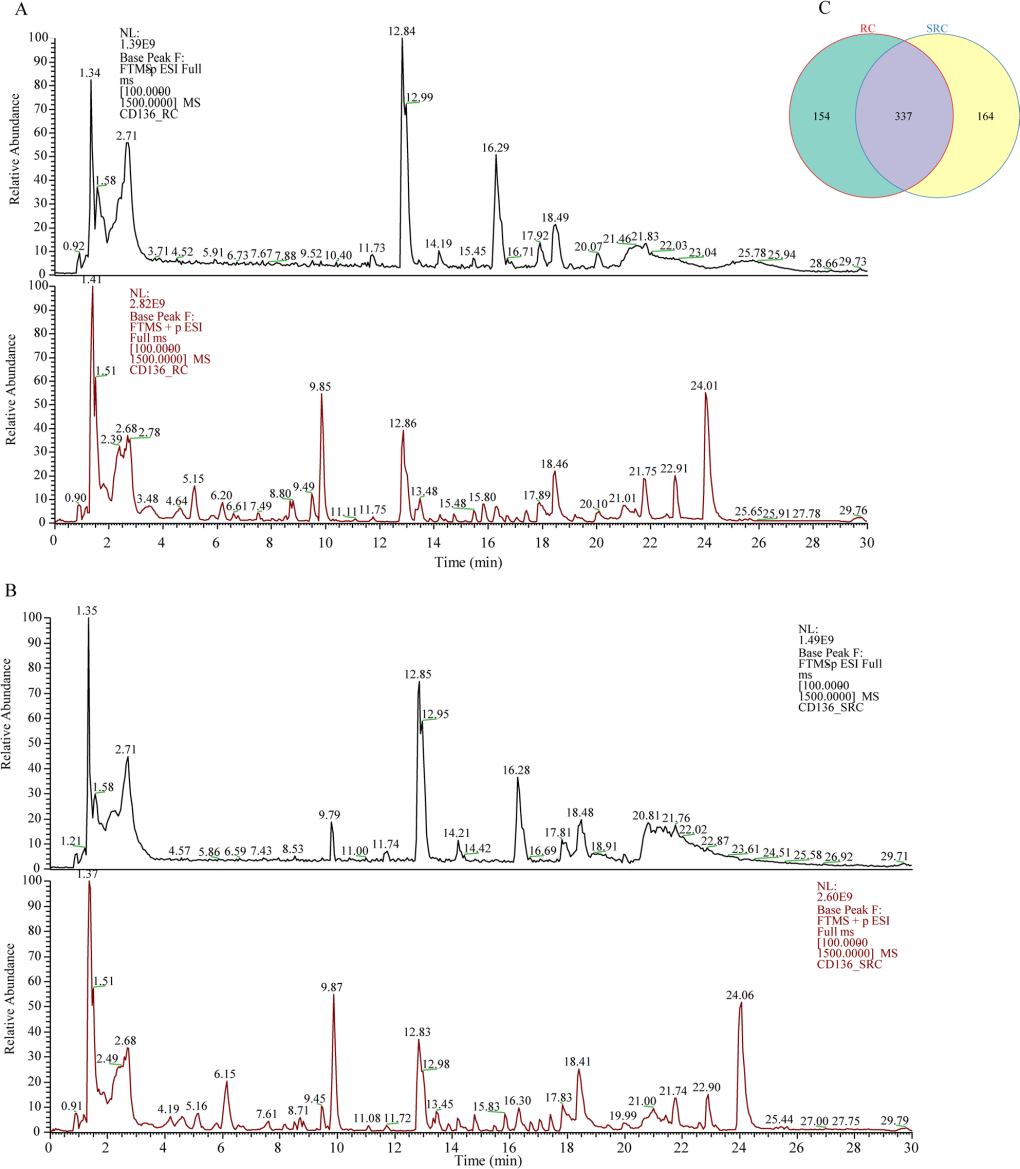
Chemical Component Analysis of RC and SRC by UPLC-MS. (**A**) Chemical component profile of RC. (**B**) Chemical component profile of SRC. (**C**) Venn diagram of shared components

The UPLC-MS spectra of RC had been published in our previous one (doi: 10.2174/0118715303385546250912094219) and SRC are shown in Fig. 1A. This analysis led to the identification of 491 compounds in RC with matches in the mzCloud database, among which 349 compounds had an overall score greater than 60. The analysis of SRC led to the identification of 501 compounds with matches in the mzCloud database, among which 357 compounds had an overall score greater than 60. A Venn diagram analysis indicated that there are 337 components common to RC and SRC (Fig. 1C). The top 10 components according to relative abundance in the intersection are listed in Table 1. Among them, diosgenin and oleanolic acid are considered to be marker components of RC and SRC.

### The effects of RC and SRC on body weight and lipid profiles in AS mice

**Fig. 2:**
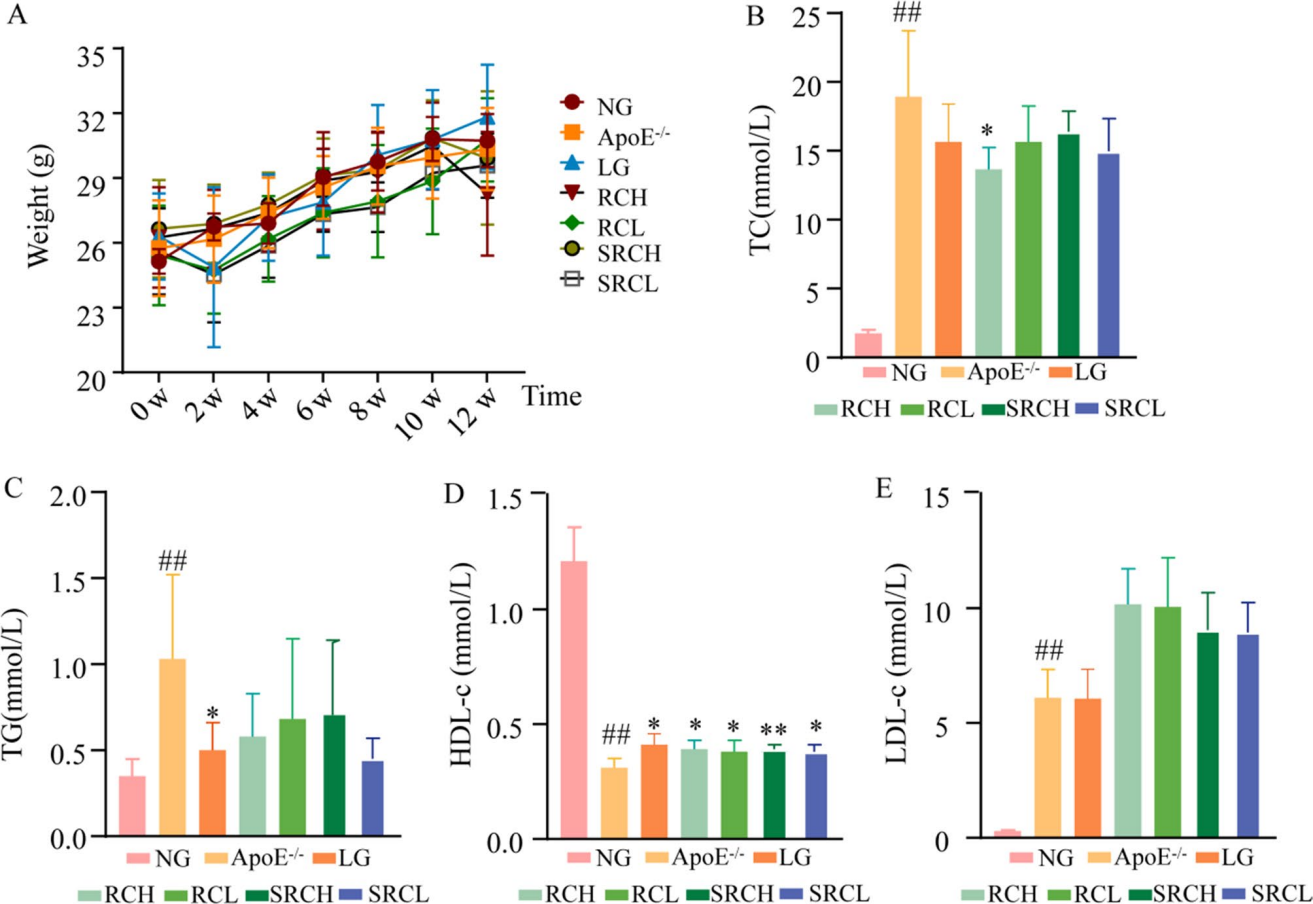
The effects of RC and SRC on body weight and lipid profiles in a murine model of AS. (**A**) Mouse body weights after the 12-week period. (**B-E**) Serum lipid profiles of mice at 12 weeks (*n* = 6):TC (**B**), TG (**C**), HDL-c (**D**), and LDL-c (**E**). Compared with NG:^#^*p* < 0.05 and ^##^*p* < 0.01. Compared with MG: *p < 0.05 and **p < 0.01

ApoE^−/−^ mice were used as a model of AS. The results of a body weight analysis following a 12-week period in which mice were fed a high-fat diet and treated with RC, SRC, atorvastatin, or saline are shown in Fig. 2A. After the 12-week period of treatment with normal saline, the body weights of model mice (group MG) and wild-type mice (NG) were not significantly different. Similarly, compared to the MG group, ApoE^−/−^ mice administered with RC or SRC for 12 weeks did not exhibit significantly different body weights.

Comparisons of serum lipid levels are shown in Fig. 2B-E. Compared with NG mice, MG mice exhibited a significant decrease in HDL levels (*p* < 0.01) and significant increases (*p* < 0.01) in levels of LDL-C, TC, and TG. Compared with MG mice, model mice treated with either dose of RC had significantly increased HDL levels (*p* < 0.05), as did model mice treated with either dose of SRC (*p* < 0.01). Moreover, mice in group RC-H experienced significantly decreased TC levels relative to group MG (*p* < 0.05), while TC levels were not significantly different in groups RC-L, SRC-H, or SRC-L as compared with MG. No significant differences were observed in the levels of LDL-C or TG upon treatment with RC or SRC (Fig. 2C, E).

### The effects of RC and SRC on vascular histopathology in AS mice

**Fig. 3:**
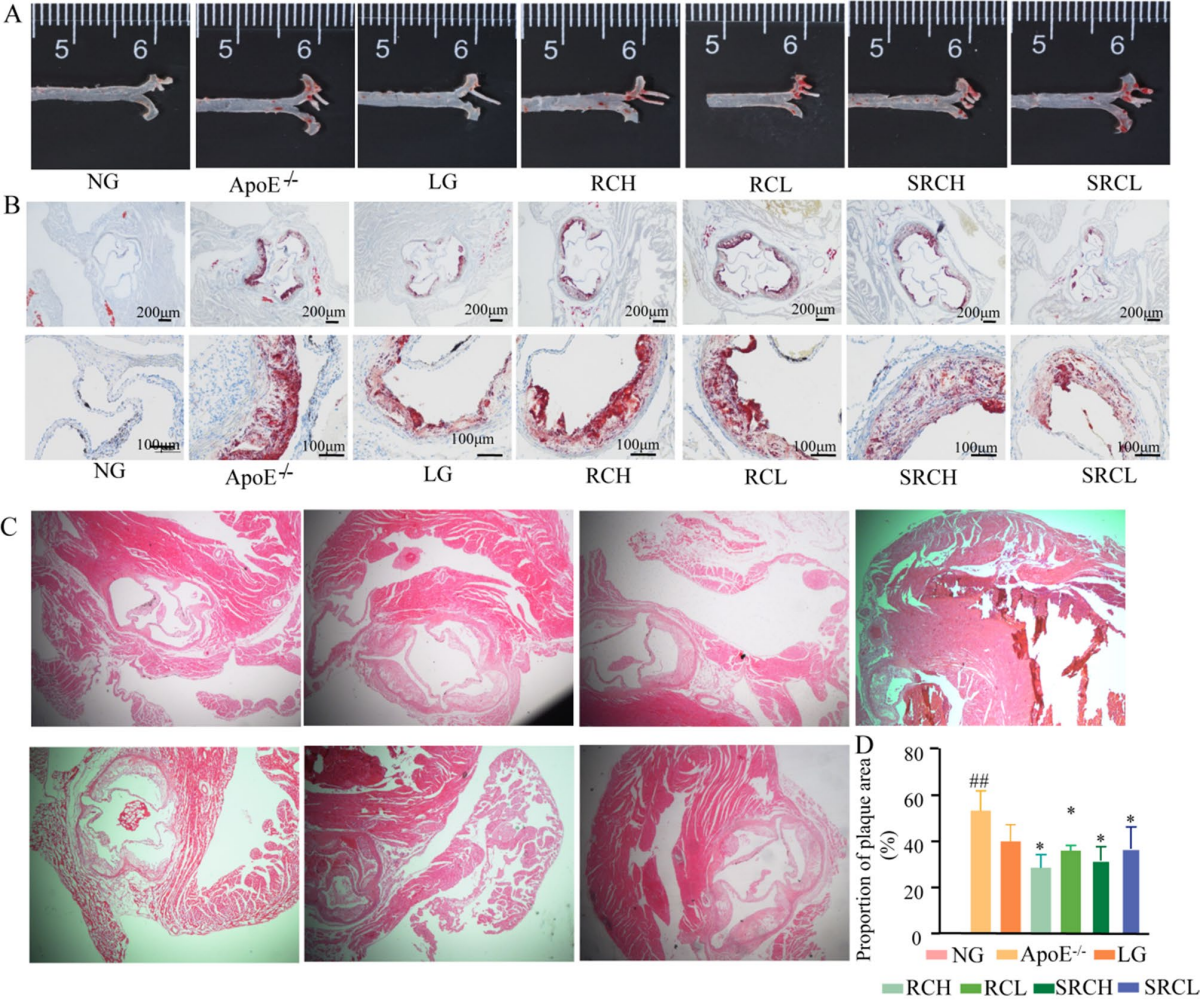
RC and SRC attenuate atherosclerotic lesions in AS mice. (**A**) Light microscopy ofplaque accumulation. (**B**) Oil red O staining of aortic arch (magnification 40×, 200×). (**C**) H&E staining of aortic arch. (**D**) Plaque area quantification (*n* = 3). Compared with NG: ^#^*p* < 0.05 and ^##^*p* < 0.01. Compared with MG: *p < 0.05 and **p < 0.01

As shown in Fig. 3A, administration of a high dose of RC (RC-H) to ApoE^−/−^ mice significantly reduced the number and volume of atherosclerotic plaques in aortic tissues. Oil Red O staining of cells in the aortic root (Fig. 3B) revealed no lipid plaques in the aortic sinus of NG mice, while MG mice had severe lipid deposition in plaques, as evidenced by large areas of red staining. However, administration of either RC or SRC improved these Oil Red O-positive lesions.

H&E staining (Fig. 3C, D) showed that in the aortas isolated from mice of group NG, the cells were neatly arranged, with no foam cells or calcification deposits observed. In comparison, tissues isolated from MG mice exhibited markedly increased lesion areas, and the plaques had larger necrotic cores. In the MG tissues, numerous foam cells were visible beneath the endothelium, the arterial sinus intima was thickened, and structural damage and elastic fiber rupture were apparent, and the plaque area was significantly increased relative to that of NG mice a (*p* < 0.01). As compared to MG, analyses of tissues from RC- and SRC-treated mice revealed significantly decreased numbers of aortic plaques (*p* < 0.01).

### The effects of RC and SRC on complete blood count in AS mice

**Fig. 4:**
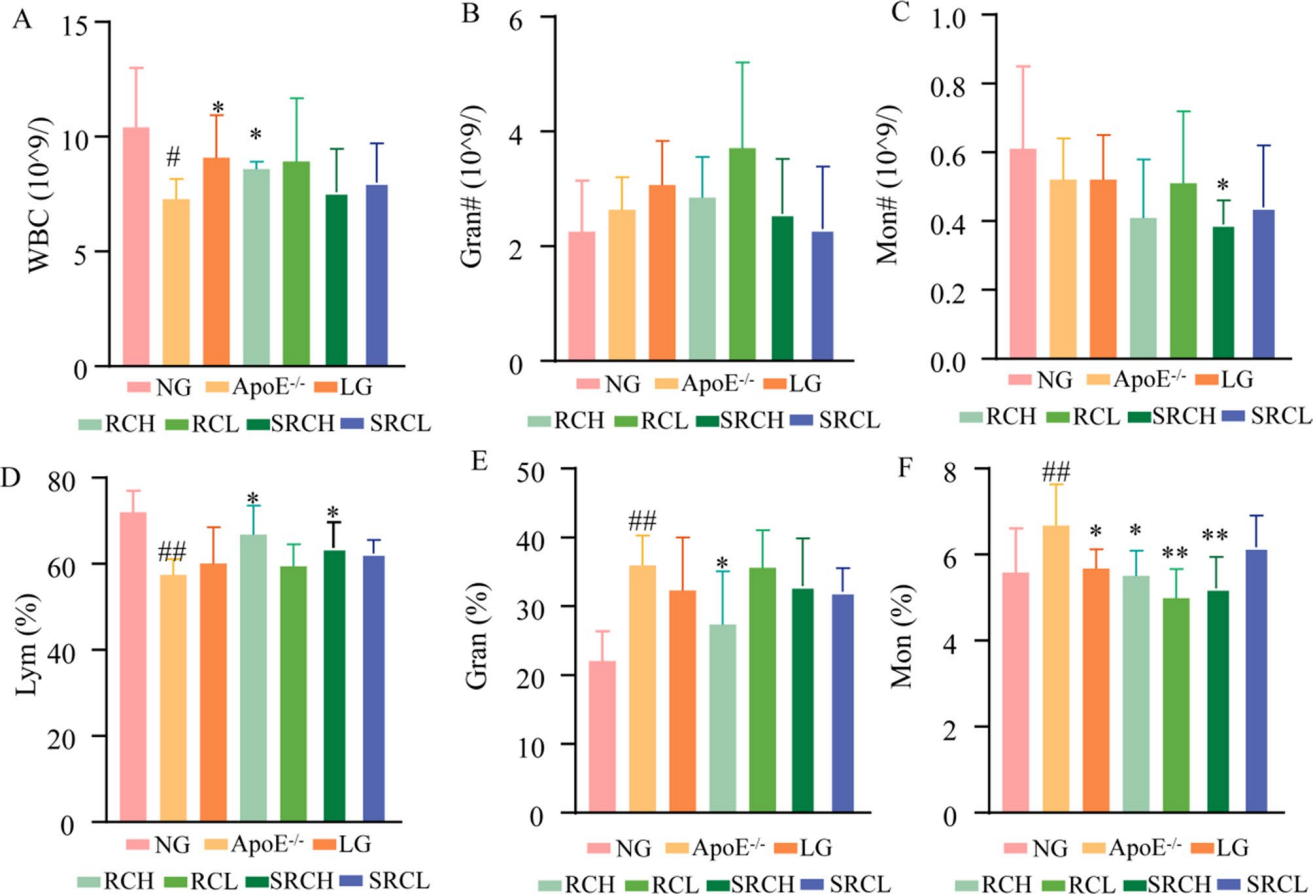
The effects of RC and SRC on blood cell numbers and distributions. (**A**) White blood cells (WBC). (**B**) Granulocyte cells. (**C**) Monocyte cells. (**D**) Lymphocyte percentage (Lym%). (**E**) Granulocytepercentage (Gran%). (**F**) Monocyte percentage (Mon%). Compared with NG: ^#^*p* < 0.05 and ^##^*p* < 0.01. Compared with MG: *p < 0.05 and **p < 0.01. *n* = 6

As shown in Fig. 4A-F, compared to NG, after 12 weeks on a high-fat diet, mice of group MG showed significantly increased of percentages of granulocyte (*p* < 0.05) and monocyte (*p* < 0.01) cells. MG mice also had significantly decreased lymphocyte percentages (*p* < 0.01) and total numbers of white blood cells (*p* < 0.01). Compared with MG, high-dose RC administration significantly reduced the percentages of granulocytes and monocytes and significantly increased the lymphocyte percentage and the total white blood cell count in model mice (all *p* < 0.05). Administration of a low dose of RC significantly reduced the percentage of monocytes (*p* < 0.01). A high dose of SRC significantly increased the lymphocyte percentage (*p* < 0.05) and significantly reduced the total number of monocytes (*p* < 0.05) and the monocyte percentage (*p* < 0.01).

### The impacts of RC and SRC on the heart function in AS mice

**Fig. 5:**
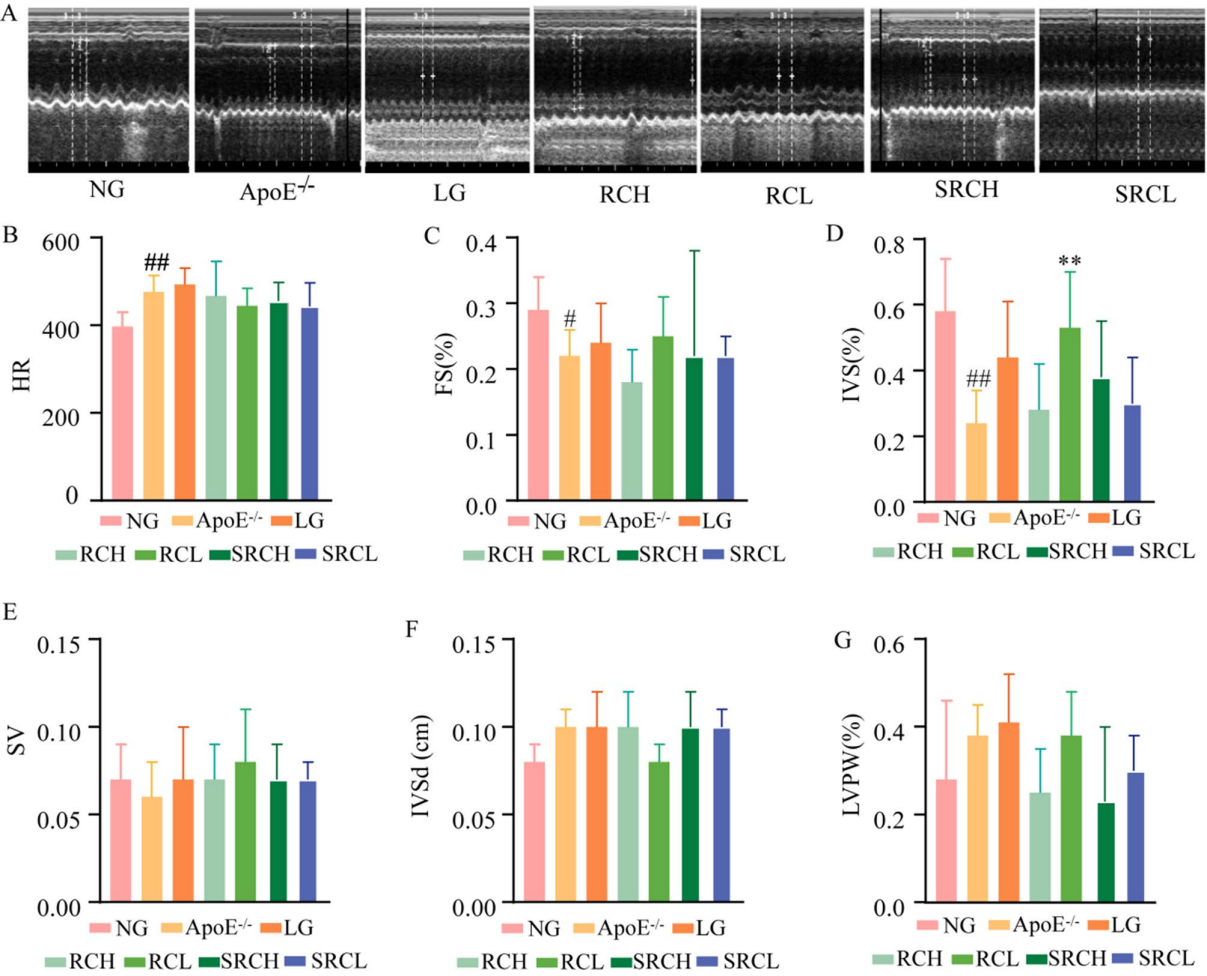
The impacts of RC and SRC on heart function in AS mice. (**A**) Echocardiograms. (**B**) Heart rate (HR). (**C**) Left ventricular short-axis shortening rate (FS). (**D**) Interventricular septum fractional thickening percentage (IVS). (**E**) Stroke volume (SV). (**F**) Left ventricular end-diastolic inter ventricular septal thickness (IVSd). (**G**) Left ventricular posterior wall (LVPW). Compared with NG: ^#^*p* < 0.05 and ^##^*p* < 0.01. Compared with MG: *p < 0.05 and **p < 0.01. *n* = 6

According to echocardiography (Fig. 5A-G), compared with NG mice, the MG mice had significantly reduced HR, FS, and IVS (*p* < 0.01, 0.05). Compared with the MG, mice in group SRC-H had significantly increased IVS (*p* < 0.05) and significantly reduced IVSd and LVPW thickness (*p* < 0.05). These results suggested that treatment with SRC can improve heart function in AS mice.

### The impacts of RC and SRC on Ly6^+^ cell populations in AS mice

**Fig. 6:**
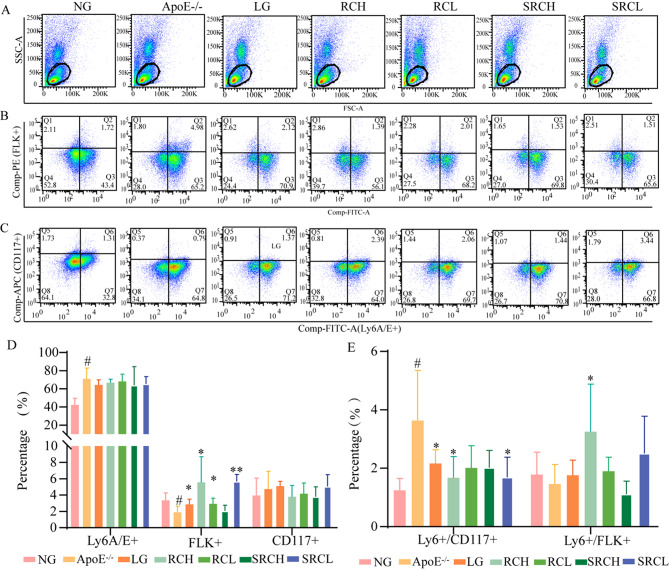
The impacts of RC and SRC on circulating progenitor cell population. (**A**) Blood lymphocytes wereidentified by forward scatter-area (FSC-A) and side scatter-area (SSC-A). (**B**) Ly6^+^/FLK^+^ cells. (**C**) Ly6^+^/CD117^+^cells. (**D**) Percentages of Ly6^+^, CD117^+^, andFLK^+^ cells. (**E**) Percentages of Ly6^+^/CD117^+^ cells and Ly6^+^/FLK^+^ cells. Compared with NG: ^#^*p* < 0.05 and ^##^*p* < 0.01. Compared with MG: *p < 0.05 and **p < 0.01. *n* = 6

In wild-type mice, a flow cytometric analysis showed that the proportion of Ly6^+^ cells among peripheral blood lymphocytes ranges from approximately 42.21% to 71.01% (Fig. 6A), while Ly6^+^/CD117^+^(Fig. 6C) and Ly6^+^/FLK^+^(Fig. 6B) cells constitute approximately 1.2% to 3.6% and 1.1% to 3.2%, respectively, of peripheral blood lymphocytes (Fig. 6). Notably, when compared to the NG group, the MG group exhibited significant increases in the proportions of Ly6^+^ and Ly6^+^/CD117^+^ cells, alongside a significant decrease in the proportion of FLK^+^ cells (all *p* < 0.05). Relative to the MG group, groups RC-H, RC-L, and SRC-L had significantly elevated proportions of FLK^+^ cells (all *p* < 0.05). Furthermore, treatment with a high dose of RC (group RC-H) was found to significantly enhance the proportion of Ly6^+^/FLK^+^ cells (*p* < 0.05), while both RC-H and SRC-L groups had significantly reduced proportions of Ly6^+^/CD117^+^ cells (both *p* < 0.05).

### Comparison of the efficacies of RC and SRC by comprehensive weight analysis

In this study, Based on clinical detection indicators for AS, this study defines TC, TG, HDL, LDL, and plaque area as I-level indicators; Gran%(%), Lym%(%), Mon%, WBC, Mon count, HR, FS, IVS, SV, IVSD, and LVPW% as II-level indicators. Based on the primary and secondary indicators and equations, if P value was greater than or equal to 2.32%, the comprehensive weight grade was considered “good”; if P was between 1.63 and 2.32%, the comprehensive weight grade was considered “medium”; if P was less than 1.63%, the comprehensive weight grade was considered “average”.

Further calculations revealed that the weighted scores (P value) associated with the RC-H, RC-L, SRC-H, and SRC-L groups were 2.15, 1.43, 1.69, and 1.57, respectively. Accordingly, the efficacy level of RC-H and SRC-H were categorized as medium, RC-L and SRC-L categorized as “average”. According to the weighted scores, we determined that RC-H was the most effective treatment, followed by the high dose of SRC and the lower doses of SRC and RC.

### Evaluating spectrum-effect relationships to identify pharmacologically active components of RC and SRC

**Fig. 7:**
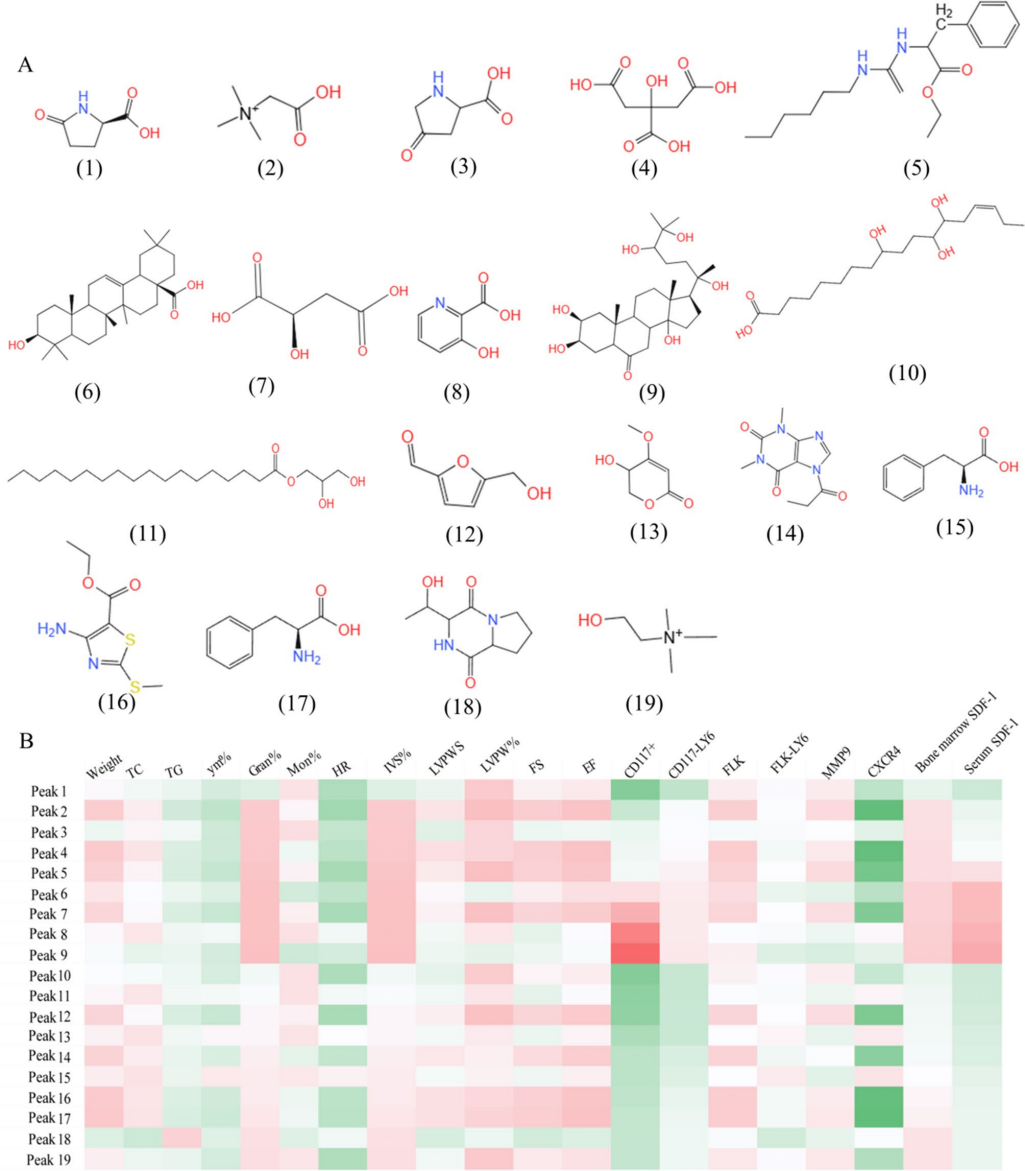
Dissecting the pharmacological effects of RC and SRC components by application of the spectrum-effect relationship. (**A**) Structural formulasof the most abundant 19 compounds found to be common between RC and SRC. (**B**) Grey relational analysis determining correlations between abundances of common ingredients and indicators of pharmacological efficacy

Our UPLC-MS analyses initially identified 337 compounds that are common to RC and SRC. Among these compounds, we identified 19 that had relative abundances of greater than 0.5% according to peak area. The chemical structures of these 19compounds that are abundant in both RC and SRC are shown in (Fig. 7A), and additional details are provided in Table 1.

**Table 1:** The top 19 peak area relative abundance in RC and SRC

Peak name	Name	Formula	m/z	RT[min]
Peak 1	D-(+)-PyroglutamicAcid	C_5_H_7_NO_3_	130.04996	2.701
Peak 2	Betaine	C_5_H_11_NO_2_	118.08639	1.405
Peak 3	4-Oxoproline	C_5_H_7_NO_3_	128.03386	2.688
Peak 4	Citricacid	C_6_H_8_O_7_	191.01863	2.712
Peak 5	ethyl2-{[(hexylamino)carbonyl]amino}-3-phenylpropanoate	C_18_H_28_N_2_O_3_	321.21646	9.861
Peak 6	Oleanolicacid	C_30_H_48_O_3_	439.35614	21.029
Peak 7	D-(+)-Malicacid	C_4_H_6_O_5_	133.01279	1.596
Peak 8	3-Hydroxypicolinicacid	C_6_H_5_NO_3_	140.03429	2.828
Peak 9	Ecdysterone	C_27_H_44_O_7_	481.31467	12.957
Peak 10	(15Z)-9,12,13-Trihydroxy-15-octadecenoicacid	C_18_H_34_O_5_	329.23279	16.303
Peak 11	Oleanolicacid	C_30_H_48_O_3_	439.35596	18.458
Peak 12	5-Hydroxymethyl-2-furaldehyde	C_6_H_6_O_3_	127.0392	5.827
Peak 13	5-hydroxy-4-methoxy-5,6-dihydro-2 H-pyran-2-one	C_6_H_8_O_4_	145.04962	4.622
Peak 14	1-Stearoylglycerol	C_21_H_42_O_4_	381.29666	22.912
Peak 15	Theophylline	C_7_H_8_N_4_O_2_	179.05493	1.352
Peak 16	L-Phenylalanine	C_9_H_11_NO_2_	166.08623	5.155
Peak 17	ethyl4-amino-2-(methylsulfanyl)-1,3-thiazole-5-carboxylate	C_7_H_10_N_2_O_2_S_2_	219.0264	1.323
Peak 18	α,α-Trehalose	C_12_H_22_O_11_	377.08481	1.453
Peak 19	2-Hydroxyphenylalanine	C_9_H_11_NO_3_	182.08127	2.064

A grey relational analysiswas performed in order to investigate correlations of the abundances of the 19 compounds with indicators of AS (Fig. 7B). We considered a relationship between a compound’s peak area and the value of an indicator of AS to be correlated if the correlation coefficient representing the relationship was greater than 0.6. Using this criterion, we identified multiple correlations. In terms of gross and biochemical indicators of AS, we determined that the area of peak 3 was correlated with effects on gain of body weight. Similarly, the areas of peaks 1, 9, 18, and 19 correlated with serum TC. In fact, the areas of all peaks except for peak 18 were found to be correlated with serum TG levels.

With respect to blood cell counts, the areas of a total of 15 peaks (1–7, 9, 10, 12, 14, and 16–19) correlated with lymphocyte abundance. Peaks 1 and 10 were correlated with granulocyte abundance. The areas of 7 of the shared peaks (4, 6, 9, 14, and 16–18) correlated with monocyte abundance.

For heart performance indicators, the areas of 16 peaks (1–10, 12, 14, and 16–19) correlated with HR, while the areas of peaks 1 and 10 correlated with IVS%. The areas of peaks 1, 3, 8–11, 18, and 19 correlated with LVPW, while the areas of peaks 6, 9, and 18 correlated with LVPW%. The areas of peaks 3, 8, 9, 11, 13, 15, and 18 correlated with the left ventricular short-axis shortening rate (FS), and the areas of peaks 3 and 18 correlated with EF.

The areas of peaks 1 and 10 through 12 correlated with the bone marrow SDF-1 level, while the areas of peaks 1 through 3 and 10 through 19 correlated with the serum SDF-1 level. The areas of 14 total peaks (1–4 and 10–19) correlated with the relative abundance of CD117^+^ lymphocytes.

### The impact of RC and SRC on the SDF-1/CXCR4 axis in AS mice

**Fig. 8:**
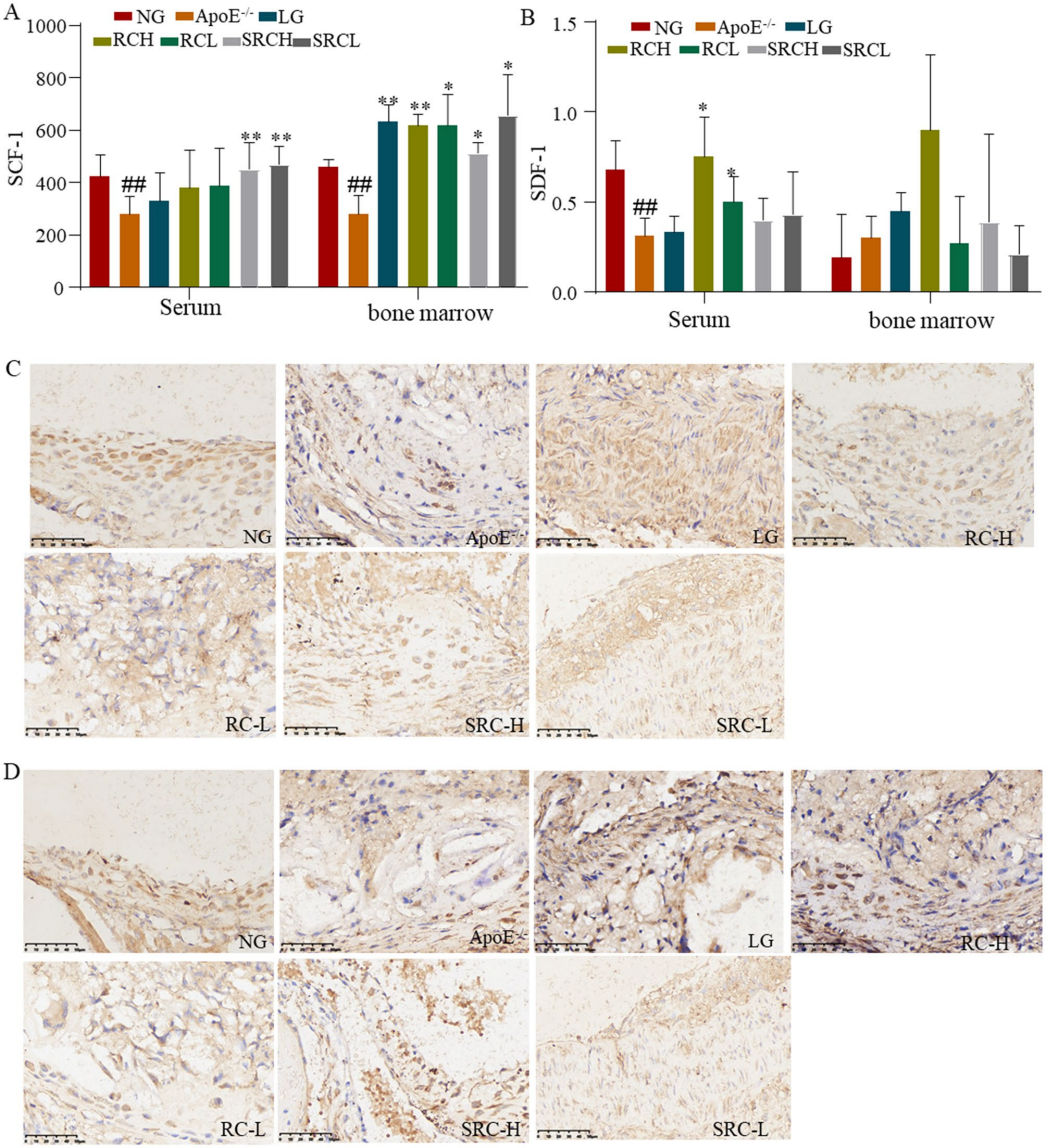
The effects of RC and SRCon the SDF-1/CXCR4 axis in AS mice. (**A**) The levels of SDF-1 in the serum and bone marrow fluid isolated from mice. (**B**) The levels of SCF-1 in the serum and bone marrow fluid isolated from mice (*n* = 6). (**C**,** D**) The levels of expression of SDF-1 (**C**) and CXCR4 (**D**) in the thoracic aorta were determined by immunohistochemistry. Compared with NG: ^#^*p* < 0.05 and ^##^*p* < 0.01. Compared with MG: *p < 0.05 and **p < 0.01

After 12 weeks of modeling on a high-fat diet with control treatments, serum SDF-1 and SCF-1 levels were significantly decreased in model mice (group MG) as compared with group NG(*p* < 0.01), but administration of high or low doses of RC (groups RC-H and RC-L, respectively) led to significantly increased serum SDF-1 levels in ApoE^−/−^ mice relative to MG control treatments (*p* < 0.05). Administration of high or low doses of SRC (SCR-H and SRC-L, respectively) significantly increased the serum level ofSCF-1 (*p* < 0.05), shown in Fig. 8A and B.

Compared with NG, after 12 weeks of modeling, the level of SCF-1 in the bone marrow supernatant from mice in group MG was significantly reduced (*p* < 0.01), while the level of SDF-1in this fluid showed no significant changes, shown in Fig. 8A and B. Compared with MG, RC-H, RC-L, SRC-H, and SRC-L all exhibited significantly increased levels of SCF-1 in the bone marrow supernatant (*p* < 0.05), shown in Fig. 8A. According to immunohistochemistry, mice in group MG had significant decreases in the levels of SDF-1 and CXCR4 in tissue from the aorta relative to the mice in group NG. Conversely, treatment with high or low RC or SRC significantly increased expression of SDF-1 and CXCR4 in the aortaas compared with group MG shown in Fig. 8C and D.

## Discussion

AS is the main pathological basis of both cardiovascular and cerebrovascular diseases. AS-related interventions can protect and stabilize atherosclerotic plaques, prevent further development of the disease, and reduce the incidence and mortality of cardiovascular and cerebrovascular diseases. Traditional Chinese medicines derived from *A. bidentata* havea multitude of effects, including removing blood stasis and dredging meridians and tonifying liver and kidney. Network pharmacology-based investigations have also found that *A. bidentata*may play exert lipid regulation and anti-inflammatory effects, thus improving vascular endothelial function, making related extracts potentially attractive multi-component, multi-target, and multi-channel treatments for AS [13].

In our research, according to the Chinese Pharmacopoeia, the recommended clinical daily dose of *A. bidentata* ranges from 5 to 12 g for an adult human (assumed body weight of 60 kg). To translate this to a murine model, we applied the body surface area (BSA) normalization method, utilizing the standard conversion factor of 12 between humans and mice. Consequently, the high dose of 2.4 g crude drug/kg was derived from the maximum clinical dose (12 g/60 kg × 12), while the low dose was set at half of that (1.2 g crude drug/kg). Thus, we chose 2.4, 1.2 g crude drug /kg as the reasonable dose selection. In this study, we demonstrated that both RC and SRC exhibit anti-AS effects in an ApoE^-/-^ mouse model, with a notably stronger efficacy observed with relatively high doses of RC. Traditionally, salt-processing is considered to strengthen the kidney-tonifying property of *A. bidentata* root in TCM theory. However, our results revealed that salt-processing could not further improve the anti-AS efficacy of *A. bidentata*. This finding provides a crucial pharmacological basis for the clinical selection of RC in the treatment of AS.

The injury and repair of the arterial vascular endothelium is a key factor during the course of AS. Accordingly, the injury and dysfunction of endothelial cells are the most important reasons for the formation of AS. In particular, the depletion of EPCs facilitates the development of AS through a loss of the ability to repair the arterial endothelium [19]. It has been shown that the number of EPCs and the number of non-aged EPCs were significantly reduced, and the activity of EPCs was significantly decreased, in patients with confirmed coronary heart disease; these reductions were directly connected to accelerated AS progression [20]. Other studies have shown that the number and function of EPCs in model animals are severely damaged in the pathological state of AS [21]. Therefore, increasing the number and function of EPCs in peripheral blood is a potential strategy to prevent and treat AS [19]. The phenotype of mouse endothelial progenitor cells (EPCs) is not defined by a single, universally accepted combination of markers, and this field is still evolving. Ly6A/E was widely used to define and sort hematopoietic stem cells (HSCs) and mesenchymal stem cells/stromal cells (MSCs) in mice. CD117 (c-Kit) has been established as a stem/progenitor cell marker in various tissues of mice [22, 23]. Ly6A/E (Sca-1) was often used in combination with other surface markers (such as c-Kit, Lin, etc.) to enrich for progenitor cell populations with stem cell-like characteristics. The Lin⁻Sca-1⁺c-Kit⁺ phenotype is used to identify hematopoietic stem cells and multipotent progenitors in mice [24]. FLK1 (VEGFR2) is a key receptor for VEGF signaling and is considered one of the early markers for endothelial and hematopoietic lineage orientation [25]. Others used flow cytometry to detect the number of EPCs in the mouse model of AS by quantifying Ly6^+^/CD117^+^and Ly6^+^/FLK^+^ progenitor cells [26]. We found that treatment with a relatively high dose of RC was associated with significant increases in the numbers of Ly6^+^/FLK^+^ and FLK^+^ cells in AS mice. Therefore, we conclude that RC-H may play an anti-AS role by increasing the number of EPCs.

*A. bidentata*has previously been shown to contain mainly triterpene saponins, sterones, alkaloids, and flavonoids, along with several other effective ingredients. In this study, our UPLC-MS analysis identified complex chemical profiles in both RC and SRC. We detected 491 and 501 distinct components in samples of RC and SRC, respectively, and the samples were found to share 337 common compounds. Particularly abundant compounds among these common components include ecdysterone; 4-dodecylbenzene sulfonic acid; palmitoleic acid; (15Z)-9,12,13-trihydroxy-15-octadecenoic acid; oleanolic acid; isovanillic acid; and 5 α-androstane-3,6,17-trione. Our results are consistent with other studies that have found that these ingredients are present in extracts of *A. bidentata* and that they are the main active ingredients leading to its anti-osteoporosis and anti-oxidative stress activities [27, 28].

In this study, we used lipid levels, numbers of arterial plaques, cardiac function, numbers of EPCs, and SDF-1 levels as indicators in a comprehensive efficacy evaluation of the anti-AS activity of *A. bidentata* root. The fact that the overall anti-AS effect of a high dose of RC was greater than that of the equivalent dose of SRC. A gray correlation analysis using these criteria found that peak 1 from the UPLC-MS analysis, which represented the compound D-(+)-pyroglutamic acid, correlated strongly with serum TC and with bone marrow and serum levels of SDF-1, suggesting a potential link between compound and the lipid-lowering and pro-stem cell homing effects observed with RC and SRC. However, these correlations do not establish causality and warrant further experimental validation. Additionally, an interesting divergence observed in our study is that RC has more comprehensive effects on systemic conditions, SRC showed better effect on improving cardiac functional parameters of IVS. The salt-processing procedure may enhance the beneficial effect on myocardial structure and function. This effect differentiation highlights the potential of herbal processing to tailor the therapeutic profile of a medicinal material towards specific pathological links within a complex disease like AS.

An axis composed of SDF-1 and its receptor CXCR4 has recently been shown to play an important role in promoting the occurrence and development of AS [29, 30]. The literature indicates that CXCL12 promotes the progression of atherosclerosis [31]. SDF-1 inhibits ABCA1-dependent cholesterol efflux from macrophages by binding to CXCR4, thereby promoting atherosclerosis [32]. The role of the CXCL12/CXCR4 axis in atherosclerosis involves functional heterogeneity and complex phenomena, and remain controversial [33]. Others reported that the SDF-1/CXCR4 axis is critically involved in the homing of progenitor cells to sites of vascular injury, promoting endothelial repair, and attenuating the progression of AS [34]. SDF-1 plays a crucial role in the mobilization of stem cells and progenitor cells, as high levels of SDF-1 retain cells within the bone marrow [35]. Clinically, granulocyte colony-stimulating factor (G-CSF) has been used to regulate SDF-1 and induce stem cell recruitment [36]. Injection of SDF-1 promotes the recruitment of EPCs, thereby accelerating the healing process of vascular injuries [37]. CXCR4 expression has been shown to be lower in non-plaque regions compared with the endothelial regions of progressive plaques, especially in proliferative endothelial regions where vascular permeability and monocyte recruitment are both increased [38]. In vascular-related physiological and pathological processes, the SDF-1/CXCR4 axis can regulate the migration and homing of EPCs to damaged blood vessels [39]. Importantly, an *A. bidentata* extract has been shown to promote stem cell migration by regulating the expression of CXCR4 [40]. Ecdysterone, palmitoleic acid, oleanolic acid, and other components that we discovered in RC and SRChave been found to improve cardiovascular disease and decrease AS progression [41, 42]; in particular, oleanolic acid has also been shown to regulate the SDF-1/CXCR4 axis [34].

Our findings indicate that RC and SRC significantly upregulate the expression of SDF-1, SCF-1, and CXCR4 in the mouse aorta as compared to control treatments, suggesting that this activity might serve as an important mechanism underlying the anti-AS activities of these formulations. The strong correlation of several of the UPLC-MS peaks, especially peaks 10 through 19, with serum SDF-1 levels. This finding provides a novel mechanistic insight into how this traditional Chinese medicine, particularly in its raw form, contributes to vascular protection. Traditional Chinese medicine principles hold that salt-processing enhances the kidney-tonifying but the processing-mediated enhancement might does not extend to anti-AS efficacy. Previous pharmacological studies on *A. bidentata* roothave focused on its anti-inflammatory and anti-osteoporosis effects [43]. However, its role in AS via modulation of the SDF-1/CXCR4 axis has not been previously reported, highlighting the novelty of our work.

## Conclusions

In summary, our results demonstrate that raw *A. bidentata* root possesses stronger anti-AS effects than its salt-processed counterpart in a mouse model. The effect is associated with specific chemical components and is likely mediated through the upregulation of the SDF-1/CXCR4 pathway. These findings provide a scientific rationale for the traditional clinical use of RC in managing AS and emphasize the need for function-specific evaluation of herbal processing techniques.

Several limitations should be acknowledged. The comparisons between groups were performed using unpaired t‑tests or ANOVA with LSD‑t post‑hoc tests without correction for multiplicity. Although consistent with many preliminary animal studies, the sample size was relatively small. Plaque area quantification *n* = 3 was insufficient to reliably detect moderate effect sizes. Although the trend of this result is consistent with the experimental results of *n* = 6 in this study, avoid over-interpretation of the statistical results. Second, the exact identities of the UPLC-MS peaks utilized in the correlation analyses (e.g., peaksD-(+)-Pyroglutamic Acid, α,α-Trehalose, and 2-Hydroxyphenylalanine) remain to be confirmed through isolation and functional validation. In future studies, these compounds should be purified and the effects of the purified components should be evaluated in vitro and in vivo. In this study, the Ly6^+^/CD117^+^and Ly6^+^/FLK^+^ defined as EPCs were not universally accepted definitive markers for EPCs in mice, more experiments were need to verified these define. Although the SDF-1/CXCR4 pathway was implicated, other mechanisms such as anti-inflammatory action or regulation of lipid metabolism may also be involved and warrant further investigation. Finally, clinical studies are needed to confirm the superior efficacy of RC in AS patients.

## Electronic Supplementary Material

Below is the link to the electronic supplementary material.

Supplementary material 1

## Data Availability

The data that support the findings of this study are available from the corresponding author upon reasonable request.

## Abbreviations

AS: Atherosclerosis
EPCs: Endothelial progenitor cells
SRC: salted Achyranthes bidentata
TCM: traditional Chinese medicine
HR: heart rate
IVS: interventricular septum fractional thickening percentage
SV: stroke volume
IVSd: left ventricular end-diastolic interventricular septal thickness
LVPW: left ventricular posterior wall thickness
EF: ejection fraction
SDF-1: stromal cell-derived factor-1
CXCR4: C-X-C chemokine receptor 4
