# Supplementary figures and images for: A comparative study on the pharmacological effects of Achyranthes root against atherosclerosis in ApoE−/− mice based on the spectrum-effect relationship
Source: Lab Anim Res. 2026 Jul 10;42:26. doi: 10.1186/s42826-026-00288-4 (PMC13352841; doi:10.1186/s42826-026-00288-4)

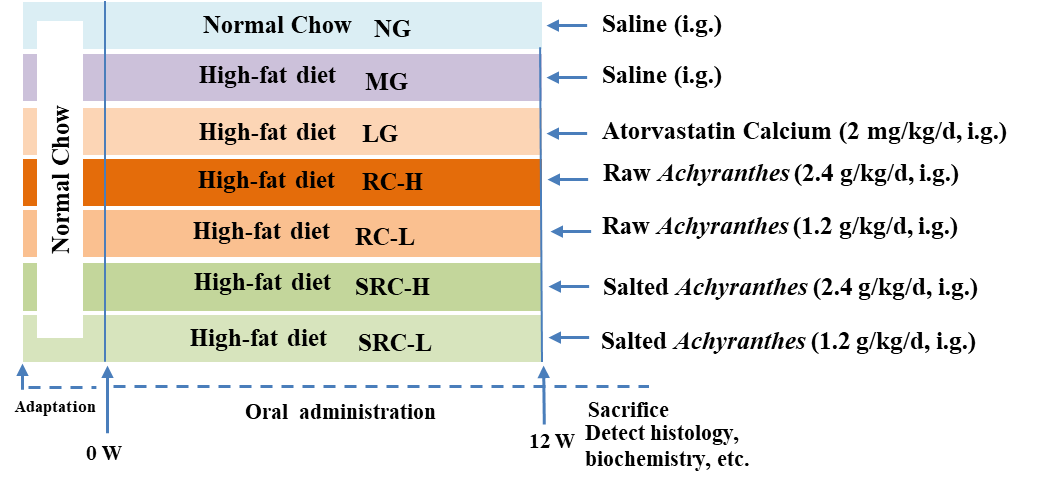


**Figure 1S****.** Summary of the experimental protocol in ApoE-/- Mice

Supplement: Supplementary file 1 — Supplementary material 1 [file 42826_2026_288_MOESM1_ESM.docx]
